# Neuroinflammation and metabolic reprogramming in Parkinson’s disease

**DOI:** 10.3389/fimmu.2026.1857394

**Published:** 2026-09-11

**Authors:** Yixin Fu, Jianghao Yu, Lu Xu, Tingting Zhou, Qingshan Deng

**Affiliations:** 1 Department of Neurosurgery, The Second People’s Hospital of Yibin, Yibin, Sichuan, China; 2 School of Medical Technology and Information Engineering, Zhejiang Chinese Medical University, Hangzhou, Zhejiang, China; 3 The First Affiliated Hospital of Zhejiang Chinese Medical University (Zhejiang Provincial Hospital of Chinese Medicine), Hangzhou, Zhejiang, China

**Keywords:** metabolic reprogramming, microglia, neuroinflammation, Parkinson’s disease, α-synuclein

## Abstract

Parkinson’s disease (PD) is the second most prevalent neurodegenerative disorder worldwide, characterized by progressive loss of dopaminergic neurons in the substantia nigra pars compacta (SNpc) and the pathological accumulation of Lewy bodies composed predominantly of aggregated α-synuclein (αSyn). Despite decades of progress in genetics and neuropathology, the mechanisms driving disease initiation and progression remain incompletely understood, and no disease-modifying therapy has yet demonstrated conclusive efficacy. Neuroinflammation and metabolic dysfunction have emerged as two central and mechanistically intertwined pillars of PD pathogenesis. We propose an integrative model in which these processes function not merely in parallel, but as mutually reinforcing components of a self-amplifying pathological circuit, while acknowledging that this model remains to be fully validated and that alternative causal architectures are possible. This review systematically addresses the mechanistic coupling between neuroinflammation and metabolic dysregulation in PD, covering: (1) the molecular basis of innate immune activation via DAMPs, pattern recognition receptors, and inflammasome signaling; (2) microglial metabolic reprogramming and the NLRP3/NF-κB inflammatory axis; (3) αSyn-driven innate and adaptive immune responses; (4) mitochondrial dysfunction and oxidative stress as bidirectional amplifiers; (5) the gut-brain axis as a conduit for peripheral immunometabolic disruption; (6) the AMPK/mTOR/HIF-1α molecular network integrating metabolism and inflammation; (7) sphingolipid metabolism and the GBA-lysosomal axis; and (8) translational evidence from animal models and randomized controlled trials. A concise section integrates key fluid biomarkers as clinical surrogates of the underlying mechanisms.

## 1 Introduction

Parkinson’s disease is the fastest-growing neurological disorder globally. The worldwide prevalence was approximately 6.1 million in 2016 and is projected to exceed 12 million by 2040 (1). Clinically, PD is defined by cardinal motor features—bradykinesia, postural instability, resting tremor, and rigidity—alongside a broad spectrum of non-motor features, including hyposmia, rapid eye movement sleep behavior disorder (RBD), and constipation, many of which precede the onset of motor symptoms by years to decades (2). Pathologically, PD is characterized by the selective loss of dopaminergic neurons in the substantia nigra pars compacta (SNpc) and the presence of intraneuronal Lewy bodies within surviving neurons (2–4). Current standard therapies, centered on levodopa and dopamine receptor agonists, provide effective symptomatic relief but do not halt the underlying neurodegenerative process. This therapeutic limitation highlights the need to transition from symptom-based management toward mechanism-driven interventions.

Parkinson’s disease currently lacks proven disease-modifying therapies despite an extensive research pipeline targeting multiple pathogenic mechanisms. The most intensively investigated approaches focus on α-synuclein, including antisense oligonucleotides, aggregation inhibitors, and immunotherapies; Among these, the anti-αSyn monoclonal antibody cinpanemab did not achieve its primary endpoint in a Phase II trial and the study was terminated due to lack of efficacy. For prasinezumab, while the Phase II PASADENA trial did not meet its primary endpoint, subgroup analyses revealed potential slowing of motor progression in patients with more rapid disease progression at baseline, particularly those taking monoamine oxidase B inhibitors or with diffuse malignant phenotypes. In the Phase IIb PADOVA trial, the primary endpoint was not met; however, the primary analysis showed a non-significant delay in confirmed motor progression with prasinezumab versus placebo (hazard ratio 0.84, 95% CI 0.69–1.01; p=0.066). Based on the aggregate evidence, prasinezumab has advanced into Phase III evaluation, underscoring both the challenges and the continued interest in αSyn-targeted immunotherapies for PD (2, 5, 6). Precision medicine strategies targeting specific genetic mutations show promise, particularly LRRK2 kinase inhibitors for LRRK2 mutation carriers and glucocerebrosidase modulators such as ambroxol for GBA mutation carriers (7–9). Repurposed drugs, including the GLP-1 receptor agonist exenatide, are also under investigation, whereas several candidates (nilotinib, inosine, isradipine, and simvastatin) have failed in clinical trials (2, 9, 10). Collectively, these findings suggest that PD represents a heterogeneous disease spectrum, in which diverse triggers converge on shared pathogenic pathways, including α-synuclein aggregation, mitochondrial dysfunction, impaired protein clearance, neuroinflammation, and oxidative stress.

The conceptual status of neuroinflammation in PD has undergone a fundamental shift—from an epiphenomenon secondary to neuronal degeneration to a primary and active driver of disease progression (11). Early observations by McGeer and colleagues first identified widespread microglial activation in the PD substantia nigra, laying the foundation for subsequent mechanistic investigations. Over the past decades, converging evidence from neuroimaging, cerebrospinal fluid (CSF) analyses, and genetic studies has firmly established neuroinflammation as a sustained and disease-defining pathological feature (2, 12, 13). *In vivo* PET imaging using TSPO (18 kDa translocator protein) ligands has demonstrated that activated microglia are diffusely distributed across multiple brain regions, including the substantia nigra, striatum, and prefrontal cortex, with activation levels correlating with motor symptom severity. At the genetic level, several risk genes implicated in PD, including LRRK2, GBA1, and PINK1, exhibit robust expression in immune cells, further supporting the notion that neuroinflammatory processes are mechanistically embedded within PD pathogenesis rather than being merely secondary to neuronal loss (14). Collectively, these findings indicate that neuroinflammation is not a mere epiphenomenon of neurodegeneration but represents a central pathogenic process that actively contributes to disease initiation and progression.

In parallel with neuroinflammation, metabolic dysfunction has emerged as a core pathological hallmark of PD. One of the earliest and most consistently observed metabolic abnormalities is a deficit in mitochondrial complex I, the first and rate-limiting component of the electron transport chain, within the substantia nigra of PD patients. This deficit has subsequently been confirmed in peripheral tissues, including platelets, skeletal muscle, and peripheral blood mononuclear cells, indicating a systemic metabolic disturbance rather than a purely localized neuronal defect. Lysosomal dysfunction represents another critical dimension of metabolic impairment. In particular, glucocerebrosidase deficiency resulting from GBA1 mutations disrupts sphingolipid metabolism, leading to the accumulation of lipid intermediates that promote α-synuclein aggregation and impair protein degradation pathways. This creates a self-amplifying cycle linking lipid dysregulation, proteostasis failure, and neuroinflammation (15).

Furthermore, metabolomics studies have revealed widespread metabolic remodeling in PD, affecting multiple biochemical pathways, including sphingolipids, phospholipids, bile acids, and short-chain fatty acids (SCFAs) in both plasma and CSF (16). These findings underscore that metabolic dysfunction in PD extends beyond energy deficits to encompass global alterations in cellular and systemic metabolic networks.

The traditional separation of inflammation and metabolism into distinct research domains has introduced significant conceptual limitations in understanding PD pathogenesis. Increasing evidence suggests that these two processes are deeply interconnected and mutually reinforcing.

**Figure 1:**
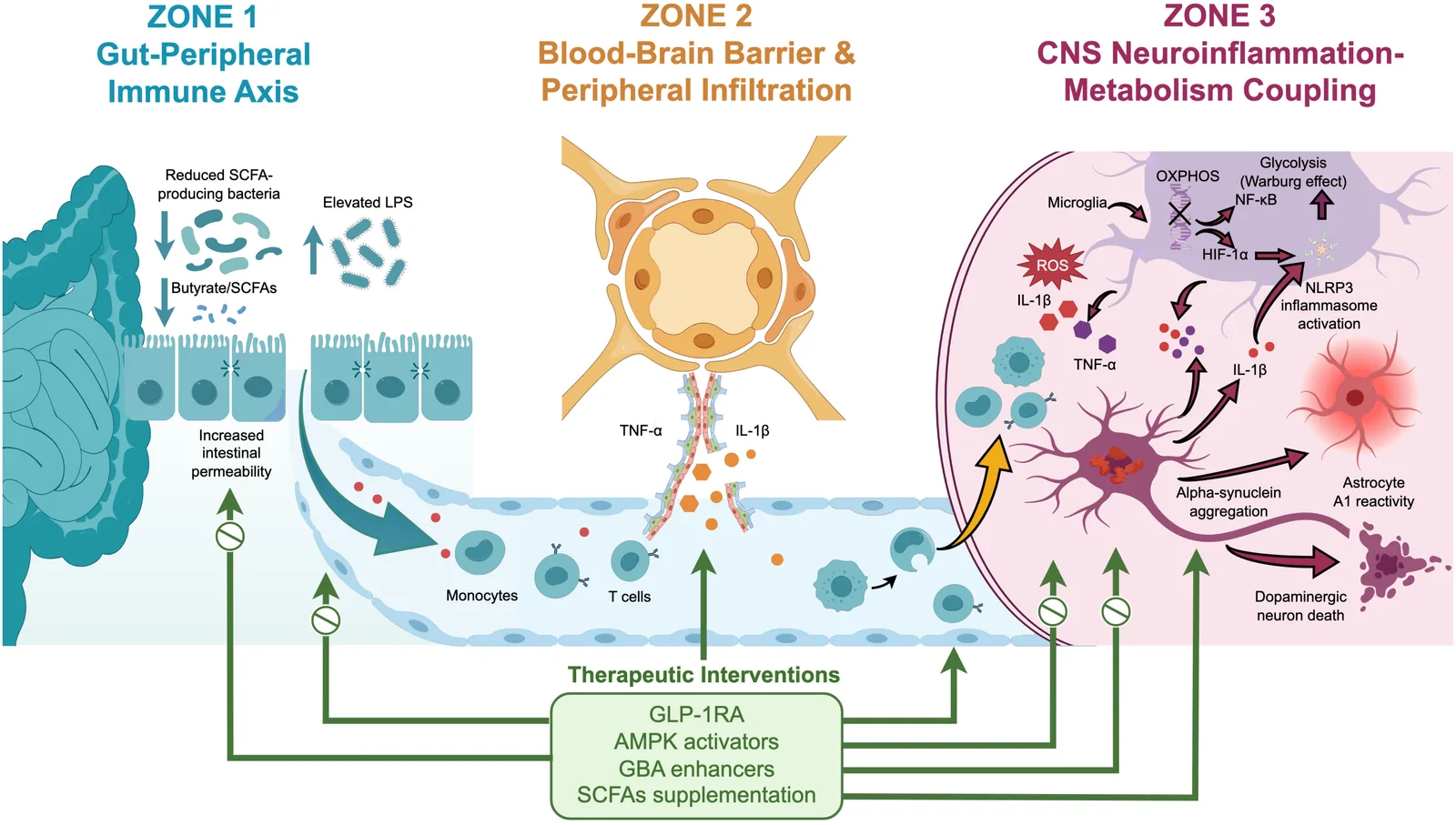
Global overview of neuroinflammation–metabolic coupling in Parkinson’s disease. The figure illustrates the full pathological axis from gut dysbiosis → reduced SCFAs → peripheral immune activation → blood-brain barrier (BBB) disruption → microglial metabolic reprogramming → αSyn aggregation → dopaminergic neuron death, along with key therapeutic intervention nodes including GLP-1 receptor agonists, AMPK activators, and GBA enhancers. Created with Figdraw.

First, microglial activation is intrinsically linked to metabolic reprogramming. The shift from oxidative phosphorylation (OXPHOS) to glycolysis is not merely a consequence of inflammatory activation but represents a fundamental prerequisite for sustaining proinflammatory responses (17). Second, mitochondrial dysfunction and oxidative stress exert broad effects across both neuronal and immune cell populations, with mitochondrial reactive oxygen species (mtROS), cytokines, and metabolic intermediates acting as shared signaling mediators that propagate pathological signals across cell types. Third, gut microbiome-derived metabolites such as SCFAs can epigenetically modulate microglial activation states, thereby directly linking peripheral metabolic disturbances to central neuroinflammation (18) (**Figure 1**).

Taken together, these observations highlight the existence of a tightly coupled immunometabolic network underlying PD pathogenesis (19–21). Integrating neuroinflammation and metabolic dysfunction into a unified mechanistic framework is therefore not merely a conceptual refinement but a translational necessity. Such an integrated perspective provides a critical foundation for the identification of early biomarkers and the development of effective disease-modifying therapies.

Therefore, this review aims to systematically elucidate the mechanistic coupling between neuroinflammation and metabolic reprogramming in PD, and to present this interaction as a coherent conceptual framework for future research and therapeutic development.

## 2 Innate immune signaling: DAMPs, pattern recognition receptors, and inflammasome integration

### 2.1 The DAMP landscape in PD

The innate immune system detects two major classes of molecular signals via pattern recognition receptors (PRRs): pathogen-associated molecular patterns (PAMPs) derived from microorganisms, and danger-associated molecular patterns (DAMPs) released from damaged or dying cells. In Parkinson’s disease (PD), neuroinflammation is predominantly sterile, driven by endogenous DAMPs rather than exogenous pathogens (14).These DAMPs arise from multiple cellular compartments and collectively contribute to sustained microglial activation.

Key sources of DAMPs in PD include proteinaceous, mitochondrial, nuclear, and lipid-derived signals. Among these, aggregated α-synuclein (αSyn) represents a central protein DAMP. Oligomeric and fibrillar αSyn species, particularly those enriched in β-sheet structures within the hydrophobic NAC domain, are recognized by TLR2 and TLR4, while oxidatively modified forms, including nitrated αSyn, exhibit enhanced affinity for TLR2 (22). Mitochondrial dysfunction further amplifies DAMP signaling through the release of mitochondrial DAMPs (mtDAMPs). These include unmethylated mitochondrial DNA (mtDNA), which is sensed by TLR9 and the cGAS–STING pathway, as well as mitochondrial transcription factor A (TFAM), cardiolipin (a known activator of NLRP3 inflammasome), and formylated peptides that engage FPR1 (23). In parallel, nuclear-derived DAMPs such as high-mobility group box 1 (HMGB1) are passively released following necrotic neuronal death and activate microglial NF-κB signaling via RAGE and TLR4, thereby linking neuronal injury to secondary inflammatory amplification (14). Lipid-derived DAMPs also contribute to this process. Lysosomal dysfunction promotes the accumulation of bioactive lipids, including ceramide and glucosylsphingosine (GluSph), while oxidative stress generates lipid peroxidation products notably 4-HNE and MDA-adducted proteins, all of which can activate TLR4-dependent signaling pathways in microglia (15, 24).

Collectively, these diverse DAMP signals converge on PRR-mediated pathways to initiate and sustain innate immune activation, ultimately providing a mechanistic bridge toward downstream inflammasome engagement and chronic neuroinflammation in PD.

### 2.2 The PRR signaling network: TLRs, NLRs, and cGAS-STING

Toll-like receptors (TLRs) represent the most extensively characterized class of pattern recognition receptors (PRRs) in PD-associated neuroinflammation and function as primary sensors of extracellular and endosomal DAMPs. Among them, TLR2 and TLR4 are particularly relevant, recognizing distinct conformations of α-synuclein as well as other DAMPs such as HMGB1. Upon activation, TLR2 and TLR4 predominantly signal through the MyD88-dependent pathway, leading to IRAK4–TRAF6–TAK1-mediated activation of the IKK complex and subsequent NF-κB nuclear translocation, thereby inducing proinflammatory gene expression. In addition, TLR4 uniquely engages the TRIF–IRF3 axis to drive type I interferon (IFN-α/β) production, highlighting its dual-pathway signaling capacity (22). Functionally, these pathways provide a critical priming signal that licenses downstream inflammasome activation.

NOD-like receptors (NLRs), particularly NLRP3, act as intracellular sensors that integrate signals downstream of TLR activation. NLRP3 inflammasome assembly requires two sequential steps: (1) a priming phase characterized by NF-κB-dependent transcriptional upregulation of NLRP3, pro-IL-1β, and pro-IL-18; followed by (2) an activation phase, triggered by cellular stress signals such as mitochondrial ROS (mtROS), potassium efflux, or lysosomal rupture, leading to NLRP3 oligomerization and ASC recruitment, thereby forming a high-molecular-weight inflammasome complex. In the effector phase, activated caspase-1 mediates the proteolytic maturation of pro-IL-1β and pro-IL-18, as well as the cleavage of gasdermin D (GSDMD), the pore-forming activity of which induces pyroptosis—a highly proinflammatory form of cell death (17). Importantly, pyroptosis constitutes a central amplification node in the PRR signaling network. The formation of GSDMD pores leads to the release of intracellular contents, including α-synuclein species, mitochondrial DNA, and lysosomal components, thereby generating a secondary wave of DAMPs that further activates neighboring microglia and propagates inflammation along neuroanatomical pathways (14).

The cGAS–STING pathway represents an additional cytosolic DNA-sensing axis that links mitochondrial dysfunction to chronic innate immune activation. The DNA sensor cGAS detects mislocalized mitochondrial DNA (mtDNA) and catalyzes the production of cyclic GMP–AMP (cGAMP), which activates the ER-resident adaptor STING, leading to TBK1–IRF3-mediated type I interferon responses as well as NF-κB signaling. Notably, in PD models with impaired mitophagy, including PINK1 or Parkin deficiency, persistent mtDNA leakage sustains chronic low-grade activation of the cGAS–STING pathway (23, 25). This pathway therefore complements TLR- and NLRP3-mediated signaling, reinforcing a feed-forward inflammatory loop.

### 2.3 Inflammasome metabolic gating: energy status determines inflammatory threshold

The threshold for NLRP3 inflammasome activation is tightly coupled to cellular metabolic status, particularly in microglia, where metabolic reprogramming critically shapes inflammatory responsiveness. In glycolysis-dominant, classically activated (M1-like) microglia, the threshold for NLRP3 activation is markedly reduced. This heightened sensitivity arises from several converging mechanisms: accumulation of the glycolytic intermediate succinate stabilizes hypoxia-inducible factor 1α (HIF-1α), thereby enhancing IL-1β transcription and providing abundant priming substrate; mitochondrial dysfunction associated with the shift from oxidative phosphorylation (OXPHOS) to glycolysis compromises mitochondrial membrane potential, with consequent sustained production of mitochondrial ROS (mtROS) as a second activation signal; and impaired OXPHOS disrupts intracellular K^+^ homeostasis, lowering the threshold for K^+^ efflux required for NLRP3 activation (17, 26, 27). Collectively, these metabolic alterations function to lower the inflammasome activation threshold and predispose microglia to exaggerated inflammatory responses.

Conversely, under metabolically intact conditions characterized by active OXPHOS, cellular energy-sensing pathways exert inhibitory control over inflammasome assembly. AMP-activated protein kinase (AMPK) functions as a key metabolic checkpoint in this regulation. Rather than directly phosphorylating NLRP3, AMPK suppresses NLRP3 inflammasome activation through multiple indirect mechanisms: it activates ULK1 to promote mitophagy, thereby reducing mitochondrial ROS (mtROS) production; it inhibits mTORC1, which alleviates metabolic pressure on the inflammasome; and it stimulates SIRT1 and PGC-1α to enhance mitochondrial biogenesis and oxidative metabolism (28, 29). By contrast, direct phosphorylation of NLRP3 at Ser295 is mediated by PKA and PKD, which exert opposing effects: PKA-mediated phosphorylation inhibits NLRP3 activation, whereas PKD-mediated phosphorylation promotes NLRP3 inflammasome assembly (22, 30). Together, these coordinated pathways establish a critical endogenous anti-inflammatory buffer that restrains inappropriate inflammasome activation.

Taken together, these findings support a model in which cellular metabolism acts as a gatekeeper of innate immune activation, dynamically tuning the threshold for inflammasome responsiveness. This “metabolic–inflammatory gate” provides a mechanistic framework for understanding disease progression, and may explain why early therapeutic intervention—when metabolic homeostasis is relatively preserved—confers greater neuroprotective benefit than interventions initiated at later stages.

### 2.4 The complement system: humoral amplification of innate immunity

The complement system, as the humoral effector arm of innate immunity, acts as a critical amplification module within the broader innate immune network in PD. Activated in response to DAMP-driven signals, complement cascades extend and reinforce PRR-mediated inflammation. In the PD substantia nigra, C1q—the initiator of the classical pathway—is secreted by reactive astrocytes and directly binds α-synuclein oligomers, leading to C3 activation and C3b-mediated opsonization of α-synuclein aggregates and synaptic structures. This process promotes complement receptor-dependent phagocytosis sucg as CRIg- and CR3-mediated, by microglia, thereby contributing to both aggregate clearance and synaptic remodeling (31, 32).

However, excessive or dysregulated complement activation exerts deleterious effects. The anaphylatoxin C5a activates microglial C5aR, amplifying proinflammatory cytokine production, while deposition of the membrane attack complex (C5b–9) on neuronal membranes induces pore formation and direct cell injury. Thus, complement activation simultaneously drives inflammatory signaling and neurodegeneration, representing a double-edged mechanism in PD pathogenesis (31, 32).

Consistent with this, elevated levels of complement activation products, including C3d, C4d, and C5a, have been detected in both cerebrospinal fluid and peripheral circulation of PD patients, where they correlate with disease severity (14). These findings indicate that complement activation not only operates locally within the central nervous system but also reflects a broader systemic component of PD-associated neuroinflammation.

## 3 Microglial activation and metabolic reprogramming: the NLRP3/NF-κB inflammatory axis

### 3.1 Microglial activation and polarization

Microglia are the resident immune cells of the central nervous system (CNS), constituting approximately 10% of all brain cells and continuously surveying the microenvironment under homeostatic conditions. In PD, microglial activation is one of the earliest and most sustained pathological features, exhibiting pronounced stage dependence and regional specificity. Rather than a uniform response, accumulating evidence indicates that microglial activation in PD is highly heterogeneous and dynamically regulated.

Although the classical M1 (proinflammatory) versus M2 (anti-inflammatory) polarization framework has been widely used, recent single-cell transcriptomic analyses have revealed a far more complex landscape. Within the PD substantia nigra, multiple microglial subpopulations coexist, including proinflammatory subsets characterized by elevated expression of IL-1β, TNF-α, and iNOS; phagocytic or disease-associated subsets marked by high expression of TREM2 and CSF1R; and dysfunctional or exhausted subsets defined by increased inhibitory receptor expression and reduced metabolic activity (14, 33, 34).

This functional heterogeneity has important implications for disease progression. Proinflammatory microglia contribute to neuronal injury, whereas phagocytically active subsets facilitate the clearance of pathological α-synuclein aggregates. However, as the disease advances, there is a progressive shift from protective, clearance-oriented phenotypes toward proinflammatory and metabolically impaired states. This transition reflects a breakdown of immune homeostasis and marks the critical tipping point from controlled to self-sustaining neuroinflammation. Notably, these distinct activation states are closely linked to differential engagement of key inflammatory pathways, including NF-κB signaling and NLRP3 inflammasome activation (33).

### 3.2 Metabolic reprogramming: the Warburg shift as inflammatory infrastructure

The coupling between microglial activation states and cellular energy metabolism—referred to as immunometabolic reprogramming—has emerged as a central paradigm in neuroinflammation. Under homeostatic conditions, microglia primarily rely on oxidative phosphorylation (OXPHOS), maintaining efficient ATP production and mitochondrial integrity. Upon stimulation by PAMPs or DAMPs, including aggregated α-synuclein, microglia undergo rapid metabolic reprogramming characterized by enhanced glycolysis and suppression of OXPHOS, adopting a Warburg-like metabolic phenotype (17).

Importantly, this transition is not merely an adjustment in energy supply but constitutes the metabolic foundation of inflammatory effector function. Glycolysis-driven metabolic rewiring promotes the accumulation of key intermediates that directly regulate inflammatory signaling. For instance, succinate stabilizes HIF-1α, thereby enhancing upregulating proinflammatory cytokines such as TNF-α and IL-1β while suppressing OXPHOS-related gene expression (26). In parallel, pyruvate kinase M2 (PKM2) acts as a critical node in metabolic–immune coupling: upon activation, PKM2 translocates to the nucleus and cooperates with HIF-1α to amplify proinflammatory gene transcription, forming a positive feedback loop between glycolysis and inflammation (17).Additionally, activation of the pentose phosphate pathway (PPP) supplies NADPH to NADPH oxidase (NOX), supporting robust superoxide (O_2_^−^) production and amplifying oxidative stress responses (26). Collectively, these pathways establish glycolysis as a central driver of the proinflammatory microglial phenotype.

Notably, under conditions of chronic α-synuclein stimulation—mimicking sustained α-synuclein seed propagation in PD—this metabolically activated state can transition into a dysfunctional or “tolerant” phenotype characterized by a collapse of both glycolysis and OXPHOS. Lu et al. demonstrated in a mouse model that such metabolic exhaustion directly impairs microglial endocytic and degradative capacity, preventing effective clearance of incoming α-synuclein aggregates and thereby accelerating pathological spread (35). These findings establish a direct mechanistic link between metabolic failure and impaired protein clearance.

### 3.3 NLRP3 inflammasome: the molecular hub of metabolic-inflammatory coupling

**Figure 2:**
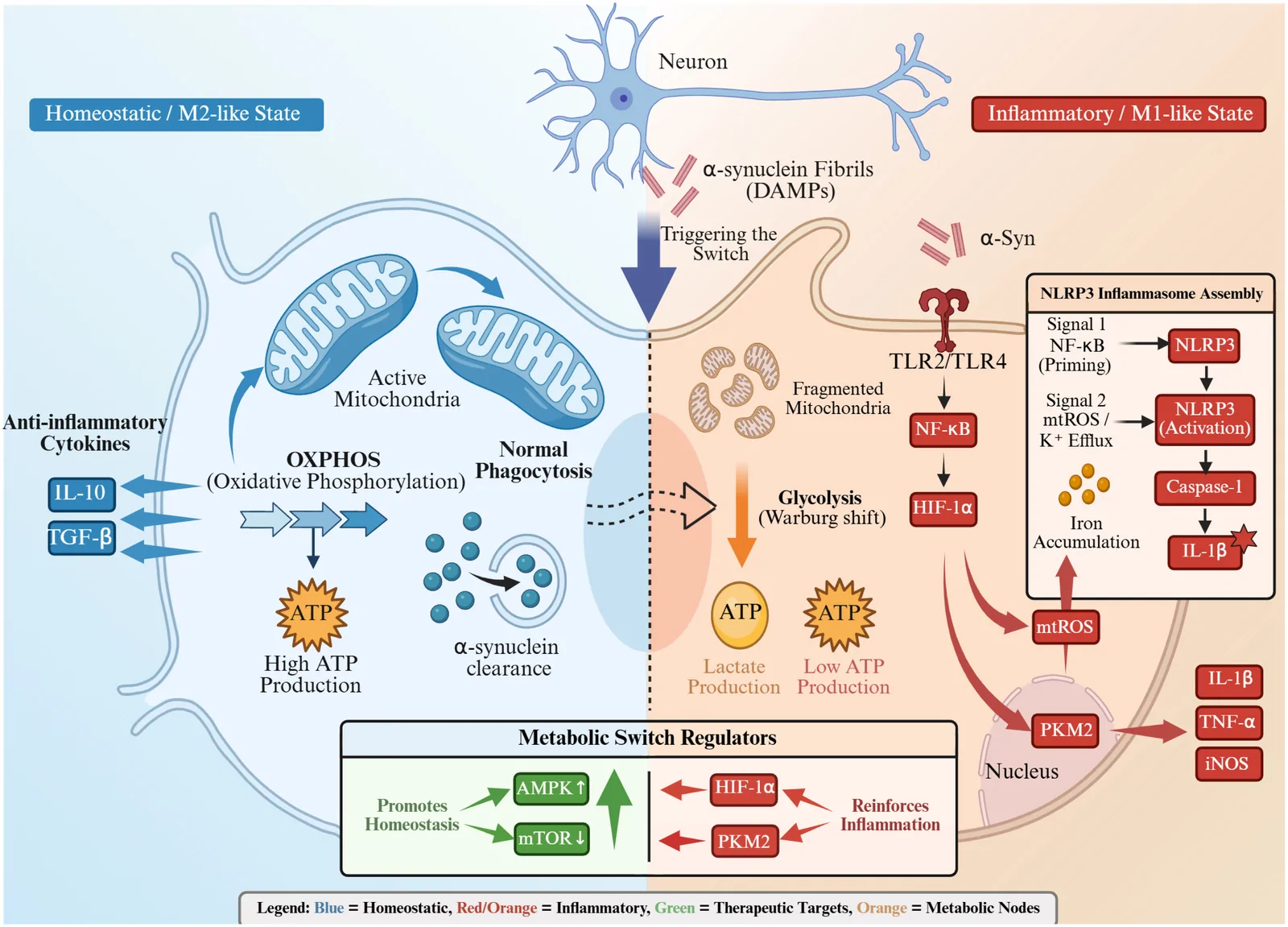
Microglial metabolic reprogramming and the NLRP3/NF-κB inflammatory axis. Left panel (blue): homeostatic/anti-inflammatory phenotype with active OXPHOS, efficient αSyn phagocytosis, and high ATP production. Right panel (red): proinflammatory phenotype with Warburg metabolic shift, NLRP3 inflammasome assembly, and massive IL-1β/TNF-α release. αSyn aggregates trigger the switch via TLR2/TLR4; the AMPK/mTOR axis determines the metabolic-inflammatory threshold. Created with BioRender.com.

The NLRP3 inflammasome is the most extensively characterized signaling complex in PD neuroinflammation (**Figure 2**). Its activation requires two signals: Signal 1 (priming: LPS or αSyn activates TLR4 → NF-κB, transcriptionally upregulating NLRP3 and pro-IL-1β) and Signal 2 (activation: mtROS, K^+^ efflux, or lysosomal damage) (17).

In the context of PD, aggregated α-synuclein uniquely contributes to both phases of inflammasome activation, acting as a dual stimulus. It engages TLR4 to provide priming signals while simultaneously inducing lysosomal destabilization to trigger activation, thereby establishing conditions for sustained NLRP3 activation. Upon assembly, the NLRP3 inflammasome recruits ASC and activates caspase-1, which processes pro-IL-1β and pro-IL-18 into their mature forms and induces pyroptosis. This process triggers the release of intracellular inflammatory mediators and damage-associated molecules, further exacerbating neuronal injury and enhancing local inflammation (36, 37).

Iron dysregulation constitutes an additional amplificatory axis within this network. Iron accumulation in the PD substantia nigra catalyzes the formation of hydroxyl radicals (·OH) via the Fenton reaction, leading to mitochondrial damage and enhanced ROS production. These oxidative signals further activate NLRP3, establishing a feed-forward loop linking iron metabolism, oxidative stress, inflammasome activation, and metabolic reprogramming toward a proinflammatory microglial phenotype (17).

Taken together, these findings position the NLRP3 inflammasome as a molecular convergence point where DAMP signaling, metabolic dysfunction, and redox imbalance intersect, driving a self-sustaining cycle of neuroinflammation in PD.

### 3.4 NF-κB: the transcriptional hub of inflammatory-metabolic integration

NF-κB (nuclear factor κB) is the master transcription factor linking inflammatory signaling with metabolic regulation. In PD, αSyn oligomers and fibrils activate the TLR2/TLR4 → MyD88 → IKK → NF-κB axis, driving large-scale transcriptional activation of IL-1β, TNF-α, IL-6, iNOS, and COX-2 (22).

At the metabolic level, NF-κB activation is deeply intertwined with metabolic reprogramming: NF-κB stabilizes HIF-1α (by suppressing PHD activity) and upregulates glycolytic enzymes (HK2, PFKFB3); conversely, glycolysis-derived succinate further stabilizes HIF-1α and primes NLRP3, while NF-κB enhances basal NLRP3 transcription. This triangular positive feedback (NF-κB ↔ HIF-1α ↔ NLRP3/glycolysis) is central to the chronification of PD neuroinflammation (38).

AMPK is the critical negative regulatory node that can break this feedback: AMPK activation simultaneously suppresses NF-κB (via IKKβ phosphorylation), inhibits mTORC1 (reducing glycolysis and anabolic metabolism), and promotes mitochondrial biogenesis (via PGC-1α), shifting microglial metabolic-inflammatory state toward a more protective phenotype (22).

### 3.5 Peripheral immune cell metabolic dysfunction: the peripheral-to-central inflammatory relay

Neuroinflammation in PD extends well beyond the CNS. A systematic review by Mark and Tansey (2025) demonstrates that PD peripheral immune cells—including monocytes, CD4^+^/CD8^+^ T cells, and NK cells—harbor significant metabolic dysfunction (23).

*PINK1* mutations impair mitochondrial OXPHOS complex I activity in patient-derived neuronal cells; *LRRK2* G2019S promotes glycolytic shift in monocytes, lowering the threshold for proinflammatory cytokine release. Critically, mitochondrial defects (reduced complex I activity) in peripheral T cells may precede CNS pathology, suggesting that peripheral immunometabolic dysregulation is an early systemic manifestation in PD pathogenesis.

The functional consequence represents a multi-mechanism escalation of CNS inflammation: metabolically dysfunctional peripheral immune cells secrete elevated proinflammatory cytokines that increase blood–brain barrier (BBB) permeability → peripheral T cells infiltrate the CNS → contact-activate local microglia → further reinforce neuroinflammation. This “periphery-to-CNS” inflammatory relay constitutes the core of the “immunometabolic axis” hypothesis (23).

Beyond metabolic dysfunction, peripheral immune cells in PD also exhibit significant functional dysregulation. A systematic analysis of PD peripheral immune cells—including monocytes, CD4^+^/CD8^+^ T cells, and NK cells—demonstrates that PINK1 mutations impair mitochondrial complex I activity and that LRRK2 G2019S promotes a glycolytic shift in monocytes, collectively lowering the threshold for proinflammatory cytokine release (23). At the CD4^+^ T cell level, Th17 cell expansion and elevated IL-17A signaling represent a consistently observed feature of PD peripheral immunity, contributing to BBB disruption and amplification of central neuroinflammation (39). CD8^+^ T cells in the context of chronic neurodegeneration display progressive functional impairment, including reduced cytotoxic efficacy and altered cytokine profiles, a pattern well-documented across neurodegenerative conditions including PD (40, 41). Importantly, mitochondrial defects in peripheral T cells may precede detectable CNS pathology, suggesting that peripheral immunometabolic dysregulation is an early systemic manifestation of PD (23). Together, these dysfunctional peripheral immune cells sustain a chronic low-grade systemic inflammatory state that increases BBB permeability, facilitates lymphocyte infiltration into the CNS, and contact-activates resident microglia, constituting the core of the peripheral-to-CNS inflammatory relay.

## 4 α-Synuclein-triggered immune responses: from protein aggregation to inflammatory cascades

### 4.1 Pathological forms of αSyn and innate immune recognition

α-Synuclein is a 140-amino acid presynaptic protein involved in vesicle trafficking and synaptic function under physiological conditions. In PD, misfolded α-synuclein aggregates into multiple pathological species, including soluble oligomers, protofibrils, and mature amyloid fibrils that constitute the core of Lewy bodies. These distinct conformational states exhibit differential immunogenicity and cytotoxic potential (15).

From an innate immune perspective, these α-synuclein species are differentially recognized by pattern recognition receptors (PRRs), establishing a direct link between protein aggregation and immune activation. Oligomeric α-synuclein, particularly when released extracellularly following neuronal membrane disruption, is preferentially recognized by TLR2, leading to MyD88-dependent NF-κB activation and proinflammatory gene transcription (8). In contrast, β-sheet-rich fibrillar α-synuclein is more effectively sensed by TLR4, which engages both TRIF- and MyD88-dependent pathways to induce combined proinflammatory cytokine and type I interferon responses (14, 22). In addition, intracellular α-synuclein species can act as a second signal for NLRP3 inflammasome activation, triggering caspase-1–dependent maturation of IL-1β and induction of pyroptosis (17, 24, 42).

These recognition events initiate a multi-layered inflammatory cascade across the CNS. Activated microglia release proinflammatory cytokines, including TNF-α, IL-1β and IL-6, and reactive oxygen species, while astrocytes undergo transformation into the neurotoxic A1 phenotype (43). Concurrently, peripheral immune cells, including T cells and monocytes, are recruited into the CNS, further amplifying the inflammatory response. Together, these processes propagate a self-reinforcing inflammatory cascade that links α-synuclein aggregation to progressive neurodegeneration.

### 4.2 Bidirectional regulation between αsyn aggregation and metabolic dysfunction

The relationship between α-synuclein (αSyn) aggregation and cellular metabolism is profoundly bidirectional, forming a dynamic and self-reinforcing interaction rather than a simple linear causality. Metabolic dysfunction creates a permissive environment that facilitates αSyn misfolding and aggregation. Oxidative stress, particularly mitochondrial-derived reactive oxygen species (ROS) such as hydroxyl radicals (·OH), induces post-translational modifications of αSyn, including nitration and nitrosylation, thereby accelerating its oligomerization and fibrillization (18). In parallel, lipid metabolic disturbances—especially sphingolipid accumulation resulting from glucocerebrosidase (GBA) deficiency—impair lysosomal function and chaperone-mediated autophagy (CMA), reducing αSyn degradation and leading to intracellular accumulation (44).

Conversely, aggregated αSyn actively disrupts multiple metabolic pathways, further exacerbating cellular dysfunction. At the mitochondrial level, αSyn interacts with outer mitochondrial membrane proteins such as TOM20 and VDAC, impairing electron transport chain activity—particularly complex I—and reducing oxidative phosphorylation efficiency (22, 45). In addition, αSyn interferes with protein clearance systems by disrupting both CMA (via LAMP-2A dysfunction) and macroautophagy (via beclin-1 inhibition), resulting in the buildup of damaged proteins and organelles (44). Furthermore, αSyn promotes an imbalance in mitochondrial dynamics by favoring excessive fission over fusion, resulting in mitochondrial fragmentation and further bioenergetic decline (44).

Collectively, these reciprocal interactions establish a pathogenic feed-forward cycle in which metabolic dysfunction accelerates αSyn aggregation, and aggregated αSyn in turn drives progressive metabolic deterioration. This self-amplifying loop represents a central mechanism underlying the acceleration of PD pathology and the transition from cellular stress to irreversible neurodegeneration.

### 4.3 Prion-like spread of αSyn and neuroinflammatory amplification

A distinctive feature of αSyn pathology is its prion-like propagation: pathological αSyn can spread from one neuron to adjacent cells via direct cell contact, exocytosis-endocytosis, and extracellular vesicles (EVs), generating the spatially progressive Braak staging pattern (46).

This propagation is tightly coupled with neuroinflammation. Extracellular αSyn aggregates activate microglia along their path, generating an “inflammatory wave” that accompanies pathological spread. Microglial clearance attempts—via phagocytosis and macropinocytosis—trigger oxidative bursts generating large quantities of ROS, potentially causing bystander injury to neighboring neurons. When microglia enter a state of “metabolic exhaustion” from chronic αSyn stimulation, their clearance efficiency is substantially reduced, accelerating αSyn spread while sustained inflammatory signals further compromise neuronal metabolic status (35).

Beyond cell-to-cell spread within the CNS, evidence supports an anterograde peripheral-to-CNS route of αSyn transmission with direct neuroimmune consequences. The Braak staging hypothesis originally proposed that αSyn pathology may initiate in peripheral sites—including the enteric nervous system (ENS) and olfactory epithelium—and propagate centrally in an anterograde manner, consistent with the early non-motor manifestations of PD such as hyposmia and constipation that typically precede motor onset by years to decades (9, 18). In the gut, PD-associated dysbiosis promotes intestinal inflammation and increased gut permeability, facilitating the exposure of ENS neurons to luminal contents and microbial metabolites that may initiate or accelerate local αSyn aggregation (18, 47). Once formed in ENS neurons, pathological αSyn species can propagate transneuronally along vagal and sympathetic projections toward the brainstem and subsequently to midbrain structures, as reflected in the hierarchical distribution of Lewy pathology across Braak stages (46). At each relay station along this anterograde route, extracellular αSyn seeds activate TLR2 and TLR4 on resident macrophages and microglia, generating local inflammatory responses that may accelerate pathological spread (14, 24). Peripheral dendritic cells primed by enteric αSyn in gut-associated lymphoid tissue can further generate αSyn-specific effector T cells that traffic to the CNS, establishing an adaptive immune cascade that amplifies the innate inflammatory response initiated at each propagation node.

## 5 Adaptive immune responses in PD: T cell subset differentiation, exhaustion, and tolerance breakdown

### 5.1 Evidence for adaptive immune activation in PD

**Figure 3:**
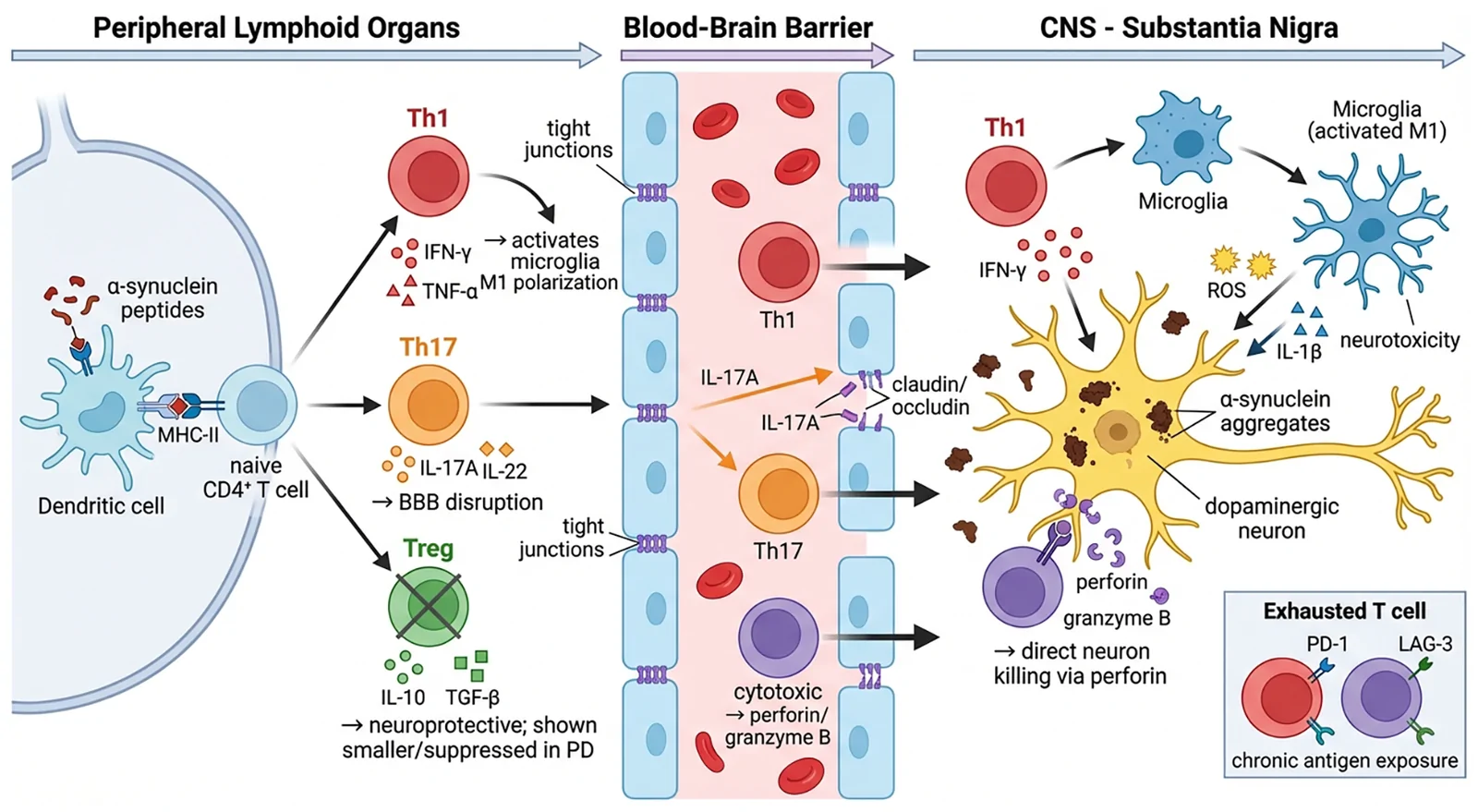
Cellular mechanisms of adaptive immune responses in Parkinson’s disease. Dendritic cells present αSyn epitopes via MHC-II, driving CD4^+^ T cell differentiation toward Th1 (IFN-γ/TNF-α → microglial M1 polarization), Th17 (IL-17A → BBB tight junction disruption), and functionally impaired Tregs (IL-10↓). CD8^+^ cytotoxic T lymphocytes infiltrate the CNS through the disrupted BBB and directly kill dopaminergic neurons via perforin/granzyme B. Chronic antigen exposure leads to T cell exhaustion (PD-1/LAG-3 upregulation). Created with BioRender.com.

PD has long been considered a disease dominated by CNS-intrinsic innate immunity (microglia), but evidence accumulated over the past decade increasingly implicates adaptive immunity—particularly T cells—as core participants in PD pathogenesis (14)(**Figure 3**). Landmark findings include: Sulzer et al. (2017, *Nature*) demonstrated, using HLA tetramer technology, αSyn epitope-specific T cell clones in PD patient peripheral blood at significantly higher frequencies than in healthy controls—direct evidence of αSyn-specific adaptive immune responses. Immunohistochemical studies have identified CD4^+^ and CD8^+^ T cell infiltration in the PD substantia nigra, striatum, and prefrontal cortex, with higher density around Lewy body-enriched regions (23). GWAS studies have identified multiple PD risk variants in the HLA region, further supporting a role for antigen presentation and adaptive immunity (48).

### 5.2 CD4^+^ T cell subset differentiation and immunometabolic coupling

CD4^+^ helper T cells exert bidirectional effects in PD neuroinflammation, which are determined by their differentiation into distinct functional subsets and tightly coupled metabolic programs. Together, these subsets form a dynamic balance between proinflammatory and regulatory arms that shapes disease progression (49).

Proinflammatory subsets are predominantly represented by Th1 and Th17 cells. Th1 cells, polarized by IFN-γ and IL-12, constitute a major infiltrating CD4^+^ population in PD. Through the secretion of IFN-γ, Th1 cells strongly polarize microglia toward a proinflammatory phenotype, enhance MHC-II expression to facilitate antigen presentation, and induce iNOS via JAK–STAT1 signaling, thereby amplifying nitric oxide–mediated neuronal injury. From a metabolic perspective, Th1 cells rely heavily on aerobic glycolysis and glutaminolysis, establishing functional and metabolic synergy with proinflammatory microglia (23).

Th17 cells contribute to disease progression through complementary mechanisms involving barrier disruption and inflammatory amplification. Cytokines, including IL-17A and IL-22, act directly on brain microvascular endothelial cells to downregulate tight junction proteins, notably claudin-5 and occludin, thereby increasing BBB permeability—a critical step enabling lymphocyte infiltration into the CNS. Elevated IL-17 levels in CSF and plasma correlate with the severity of PD patients. In addition, IL-17 activates NF-κB signaling in astrocytes and microglia, establishing a feed-forward cascade linking BBB disruption, immune cell recruitment, and sustained neuroinflammation (14, 39).

In contrast, regulatory subsets—including Th2 cells and regulatory T cells (Tregs)—mediate anti-inflammatory and neuroprotective effects. Th2-associated cytokines (IL-4, IL-10, and IL-13) promote alternative microglial activation and support neurotrophic factor production. Tregs (Foxp3^+^CD4^+^CD25^+^) function as key suppressors of excessive immune activation by maintaining immune homeostasis and limiting proinflammatory cytokine production. However, within the PD substantia nigra microenvironment, these protective responses are significantly compromised. Chronic exposure to proinflammatory cytokines such as TNF-α and IL-6 destabilizes Treg function via mTOR activation and modulation of the SIRT1–Foxp3 axis, leading to reduced suppressive capacity and diminished IL-10 secretion. Consistently, decreased Treg frequency and function correlate with disease severity and neuroinflammatory burden in PD patients (23).

Collectively, PD is characterized by a pronounced imbalance in the CD4^+^ T cell compartment, in which proinflammatory Th1/Th17 responses dominate over Th2/Treg-mediated regulation. This shift toward a proinflammatory immunometabolic state reinforces innate immune activation and contributes to the persistence and amplification of neuroinflammation.

### 5.3 CD8^+^ cytotoxic T lymphocytes: direct neuronal killing

CD8^+^ CTL-mediated neuronal injury has received increasing attention. αSyn epitopes can be presented via MHC-I on neuronal surfaces (upregulated under IFN-γ stimulation), making neurons direct targets for CD8^+^ CTL attack through perforin/granzyme B or Fas-FasL pathways (14, 50). Animal model evidence supports a direct pathological role for CTLs: dopaminergic neuron loss in αSyn PFF-injected mice is significantly attenuated in T cell-deficient (RAG^−^/^−^) backgrounds and partially restored upon CD8^+^ T cell transfer (40).

### 5.4 Immune memory and epigenetic imprinting: the chronification mechanism

Adaptive immune responses establish immunological memory to αSyn, enabling rapid reactivation even when pathological burden transiently decreases. Memory T cell maintenance depends on a metabolic switch from the high-glycolysis of initial activation to the fatty acid oxidation (FAO) required for long-term memory survival, driven by AMPK and PGC-1α (23).

Chronic antigen exposure in PD can drive T cell “exhaustion”—a state characterized by inhibitory receptor expression (PD-1, LAG-3, TIM-3), mitochondrial dysfunction, and impaired metabolic reprogramming. Exhausted T cells lose effective cytotoxic function while continuing to secrete low-level proinflammatory cytokines, creating a state of “inefficient chronic inflammation” that may explain the persistence and resistance to resolution of PD neuroinflammation (40, 41).

The metabolic basis of peripheral T cell functional decline in PD is increasingly understood. Chronic αSyn antigen exposure and the sustained proinflammatory milieu drive peripheral T cells toward progressive metabolic inflexibility, characterized by impaired mitochondrial oxidative phosphorylation and a failure to execute the metabolic transition—from glycolysis to fatty acid oxidation—required for effective long-term immune memory (23, 40). This metabolic deterioration is compounded by mTOR hyperactivation, which suppresses AMPK/PGC-1α-driven mitochondrial biogenesis and further compromises T cell functional capacity. The CD8^+^ T cell compartment is particularly affected: in the context of chronic neurodegeneration, CD8^+^ T cells exhibit reduced cytotoxic efficacy, altered cytokine secretion profiles, and features consistent with functional exhaustion observed across chronic inflammatory conditions (40). Rather than resolving neuroinflammation, these metabolically impaired T cells maintain a state of chronic low-level cytokine secretion—including TNF-α, IFN-γ, and IL-6—that sustains BBB disruption and microglial activation (41). Whether the canonical epigenetic programs underlying T cell exhaustion in chronic viral infection or cancer (e.g., PD-1 upregulation, H3K27 trimethylation at effector loci) are operant in PD-specific peripheral T cells remains an important open question requiring direct investigation in PD patient cohorts.

### 5.5 Antigen presentation and peripheral priming

Professional antigen-presenting cells—primarily dendritic cells (DCs)—are required for adaptive immune priming. In the gut, lamina propria DCs that encounter enteric αSyn (early αSyn pathology is detectable in the enteric nervous system) initiate systemic adaptive immunity via mesenteric lymph nodes, delivering activated T cells to the CNS via the circulation^14^. From an immunometabolic perspective, sphingolipid dysregulation in PD (ceramide accumulation) alters lipid raft composition, affecting TCR signaling complex assembly efficiency and MHC-peptide complex stability—directly linking lipid metabolic dysregulation to adaptive immune response efficacy (15).

## 6 Mitochondrial dysfunction and oxidative stress: the energetic core of inflammatory-metabolic coupling

### 6.1 Multi-level evidence for mitochondrial dysfunction in PD

Mitochondrial dysfunction is one of the most firmly established pathological mechanisms in PD, with evidence spanning from epidemiology to molecular mechanism.

The historical starting point was the accidental observation in the 1980s that heroin users injected with 1-methyl-4-phenyl-1, 2, 3, 6-tetrahydropyridine (MPTP) developed rapidly progressive parkinsonism; mechanistic studies revealed that its active metabolite MPP^+^ selectively inhibits mitochondrial complex I, faithfully reproducing core PD neuropathology. Subsequent studies confirmed complex I activity reduction of approximately 30% in the PD substantia nigra, with similar deficits detectable in platelets, skeletal muscle, and peripheral blood mononuclear cells (23, 36, 51).

Genetic evidence further solidifies this connection: mutations in PTEN-induced putative kinase 1 (*PINK1*) and *Parkin* (E3 ubiquitin ligase) are among the most critical genetic causes of autosomal recessive PD, and the core function of the PINK1/Parkin pathway is mitophagy, a selective quality control mechanism that eliminates dysfunctional mitochondria (23). Pathogenic *LRRK2* mutations (G2019S) are also associated with mitochondrial fragmentation and functional impairment.

### 6.2 The PINK1/parkin pathway: intersection of mitochondrial quality control and neuroinflammation

PINK1/Parkin-mediated mitophagy represents the cellular mechanism for clearing dysfunctional mitochondria and preventing the release of their harmful contents. Under normal conditions, PINK1 is continuously cleaved by proteases on the outer mitochondrial membrane; when mitochondrial membrane potential (ΔΨm) collapses (a damage signal), PINK1 accumulates on the outer membrane, phosphorylates Parkin, which ubiquitinates outer mitochondrial membrane proteins (VDAC, MFN1/2) to tag damaged mitochondria for autophagic degradation (23).

Loss of PINK1 or Parkin function prevents effective clearance of damaged mitochondria; accumulated mtDNA and oxidative stress products directly activate the cGAS-STING innate immune pathway, triggering type I interferon responses and NF-κB activation—a key molecular pathway mechanistically connecting mitochondrial dysfunction to neuroinflammation (14).

### 6.3 Mitochondrial ROS: the chemical language of inflammatory signaling

Mitochondrial reactive oxygen species (mtROS) represent key chemical mediators linking mitochondrial dysfunction to neuroinflammation. Impairment of electron transport chain complexes I and III facilitates electron leakage, resulting in superoxide (O_2_^−^) production, This reactive species is subject to two competing fates: dismutation to hydrogen peroxide (H_2_O_2_) via superoxide dismutase 2 (SOD2), or reaction with NO to yield peroxynitrite (ONOO^-^), a potent prooxidant molecule (18).

Beyond their role as byproducts of metabolic dysfunction, mtROS function as central signaling integrators that coordinate multiple inflammatory pathways. Elevated mtROS directly contribute to NLRP3 inflammasome activation as a second activation signal, driving caspase-1 activation and the maturation of IL-18 and IL-1β (17). In parallel, oxidative modifications of α-synuclein—including tyrosine nitration and methionine oxidation—enhance its propensity to aggregate and render it resistant to proteasomal and lysosomal degradation (18), thereby increasing the burden of pathogenic protein species.

mtROS also activate NF-κB signaling via redox-sensitive IKK complex activation, driving transcription of proinflammatory genes. Furthermore, oxidative injury to mitochondrial DNA (mtDNA)—which lacks protective histones—leads to its cytosolic release, where it serves as a potent DAMP to engage the cGAS–STING pathway. Through these interconnected mechanisms, mtROS establish multiple feed-forward amplification loops linking oxidative stress, protein aggregation, inflammasome activation, and innate immune signaling.

The substantia nigra in PD is especially vulnerable to mtROS-mediated damage owing to the intrinsic properties of dopaminergic neurons. Dopamine metabolism, through both monoamine oxidase B (MAO-B)–mediated degradation and spontaneous auto-oxidation, generates substantial amounts of H_2_O_2_, resulting in elevated basal oxidative stress. This intrinsic vulnerability provides a mechanistic basis for the selective degeneration of dopaminergic neurons in PD (52, 53).

### 6.4 Lipid peroxidation and ceramide: the common currency of metabolic-inflammatory pathology

Oxidative stress not only directly damages proteins and DNA through ROS but also profoundly remodels membrane lipid composition and cell signaling through lipid peroxidation. Oxidation products of polyunsaturated fatty acids (PUFAs)—4-hydroxynonenal (4-HNE) and malondialdehyde (MDA)—are bioactive lipid mediators that modify protein function and activate inflammatory signals (18).

Critically, oxidative stress upregulates ceramide synthesis: sphingomyelinase is activated in the oxidative environment, hydrolyzing sphingomyelin to ceramide, which acts as a “stress lipid messenger” promoting apoptosis and further damaging mitochondrial integrity. Ceramide levels are broadly elevated in the PD substantia nigra and plasma, with specific ceramide species, including C16:0-Cer, correlating with the degree of dopaminergic neurodegeneration^15^.

## 7 Immunometabolic molecular networks: AMPK/mTOR/HIF-1α integrative regulation

### 7.1 The triangular control hub: AMPK, mTOR, and HIF-1α

Immunometabolic coupling is not merely a process in which metabolism supplies energy for inflammation; rather, it represents a deeply integrated regulatory system in which metabolic sensors and inflammatory signaling pathways are tightly interconnected. Within this system, AMPK, mechanistic target of rapamycin complex 1 (mTORC1), and HIF-1α form a triangular control hub that governs the balance between inflammatory activation and metabolic homeostasis (22, 26).

AMPK functions as a central anti-inflammatory and metabolic checkpoint activated by increased AMP/ATP ratios. Once activated, AMPK exerts broad protective effects by coordinating multiple downstream pathways. It suppresses NLRP3 inflammasome activation indirectly by activating ULK1 to enhance mitophagy, inhibiting mTORC1, and stimulating SIRT1/PGC-1α to promote mitochondrial biogenesis (28, 29). The direct phosphorylation of NLRP3 at Ser295 is mediated by PKA and PKD, not by AMPK (30). Additionally, AMPK signaling has been shown to suppress NF-κB activation, limiting the transcriptional priming of NLRP3 and pro-IL-1β (28). In addition, AMPK stimulates mitochondrial biogenesis via PGC-1α and activates the antioxidant transcription factor Nrf2, collectively restoring oxidative phosphorylation and redox balance. In the context of PD, genetic disruption of mitochondrial quality control—such as PINK1 mutations—impairs AMPK signaling, establishing a pathogenic cascade linking mitochondrial dysfunction, reduced AMPK activity, and enhanced inflammasome activation (23).

In contrast, mTORC1 and HIF-1α act as key drivers of proinflammatory metabolic reprogramming. Hyperactivation of mTORC1 promotes the translation of inflammatory cytokines such as IL-1β and IL-6, through the 4E-BP1/eIF4E axis, suppresses autophagy leading to accumulation of damaged proteins and organelles, and skews T cell differentiation away from regulatory T cells toward Th17 phenotypes (26).

HIF-1α, which can be stabilized not only under hypoxia but also under inflammatory conditions, further reinforces this proinflammatory state. NF-κB–dependent transcription, succinate-mediated inhibition of prolyl hydroxylase (PHD), and ROS-induced PHD inactivation collectively stabilize HIF-1α, enabling the induction of glycolytic genes, including GLUT1, HK2, LDHA, and PKM2 and direct transcriptional activation of IL-1β (26).

### 7.2 TCA cycle “immune remodeling”: succinate, citrate, and α-KG as metabolic signals

Activated proinflammatory microglia undergo characteristic reprogramming of the tricarboxylic acid (TCA) cycle, often referred to as a “broken TCA” state, in which discrete enzymatic interruptions lead to the accumulation of metabolites with potent signaling functions (22).

One major breakpoint occurs downstream of isocitrate dehydrogenase, resulting in succinate accumulation. Succinate functions both intracellularly and extracellularly as an inflammatory signal. Extracellular succinate engages SUCNR1 (GPR91) on microglia and infiltrating monocytes, promoting IL-1β and VEGF production, whereas intracellular succinate inhibits prolyl hydroxylase (PHD), leading to stabilization of HIF-1α. In addition, succinate oxidation via complex II, particularly under reverse electron transfer conditions, generates substantial amounts of ROS, further amplifying inflammatory signal transduction.

A second breakpoint occurs upstream of α-ketoglutarate dehydrogenase, leading to citrate accumulation and export to the cytoplasm. Cytosolic citrate is converted to acetyl-CoA by ATP-citrate lyase (ACL), thereby linking metabolism to epigenetic and lipid remodeling processes. Acetyl-CoA fuels histone acetylation at promoters of inflammatory genes, enhancing their transcription, and supports fatty acid synthesis required for membrane remodeling in activated immune cells.

In contrast, α-ketoglutarate (α-KG) is enriched in anti-inflammatory (M2-like) microglia and exerts opposing regulatory effects. α-KG promotes PHD activity and facilitates HIF-1α degradation, while also serving as a cofactor for TET enzymes and Jumonji-domain histone demethylases to support immune tolerance–associated gene expression. Thus, the balance between α-KG and succinate—often represented as the α-KG/succinate ratio—functions as a key metabolic indicator that defines microglial inflammatory state (22).

### 7.3 NAD^+^/SIRT1 axis: integrating metabolism, inflammation, and aging

Intracellular NAD^+^ levels decline progressively with aging, a process that is further exacerbated in PD and closely associated with mitochondrial dysfunction and neuroinflammatory burden (23). As an essential cofactor for the deacetylase SIRT1, NAD^+^ serves as a key regulator of metabolic homeostasis and anti-inflammatory responses.

SIRT1 exerts broad regulatory effects by deacetylating multiple substrates involved in mitochondrial function, immune regulation, and inflammatory signaling. It activates mitochondrial biogenesis through deacetylation of PGC-1α, stabilizes regulatory T cell (Treg) differentiation via deacetylation of Foxp3, suppresses NF-κB–mediated transcription by targeting the p65 subunit, and inhibits NLRP3 inflammasome activation through direct deacetylation. Through these coordinated actions, the NAD^+^–SIRT1 axis serves as a central anti-inflammatory and metabolic regulatory pathway.

In PD, NAD^+^ depletion—driven by excessive PARP1 activation and impaired mitochondrial NAD^+^ regeneration—leads to reduced SIRT1 activity, resulting in the simultaneous loss of multiple protective mechanisms. This integrated failure links metabolic dysfunction, impaired immune regulation, and aging-related vulnerability. Importantly, supplementation with NAD^+^ precursors including nicotinamide mononucleotide (NMN) and nicotinamide riboside (NR) has demonstrated preliminary neuroprotective effects in preclinical PD models, with ongoing clinical investigations (23).

## 8 The gut-brain axis and astrocytes: peripheral metabolic signals and CNS inflammatory integration

### 8.1 The gut-brain axis as a metabolic-inflammatory bridge

The gut–brain axis has emerged as a critical interface linking peripheral metabolism with central neuroinflammation in PD. The Braak hypothesis proposes that α-synuclein pathology may originate in the enteric nervous system (ENS) and propagate retrogradely along the neuraxis via the vagus nerve (15). Consistent with this concept, PD-associated gut dysbiosis displays highly reproducible compositional shifts, characterized by a significant depletion of short-chain fatty acid (SCFA)-producing taxa, including *Faecalibacterium prausnitzii*, *Roseburia intestinalis*, and *Blautia*, alongside a relative enrichment of potentially proinflammatory taxa such as *Akkermansia* (associated with increased intestinal permeability) and Desulfovibrio (a producer of hydrogen sulfide (47).

Gut microbial metabolites serve as key mediators of this gut–brain crosstalk, with SCFAs—primarily butyrate, propionate, and acetate—playing central regulatory roles in neuroimmune homeostasis. SCFAs exert anti-inflammatory effects through multiple, interconnected mechanisms. Butyrate functions as a natural inhibitor of histone deacetylases (HDACs), promoting histone H3/H4 acetylation and suppressing transcription of proinflammatory genes such as TNF-α, IL-1β, and iNOS, thereby establishing an epigenetic link between microbial metabolism and microglial activation (18). In parallel, SCFAs activate G protein–coupled receptors GPR41 (FFAR3) and GPR43 (FFAR2) in inestinal and immune cells, attenuating proinflammatory cytokine production.

Moreover, SCFAs play a critical role in maintaining intestinal barrier integrity. Butyrate serves as a principal energy substrate for colonocytes, thereby supporting tight junction protein expression and limiting movement of microbial components such as lipopolysaccharide (LPS) into the systemic circulation (47). At the central level, SCFAs are also essential for proper microglial maturation and functional homeostasis, as demonstrated by germ-free mouse models in which SCFA deficiency leads to immature and hyperresponsive microglial phenotypes.

In addition to SCFAs, bile acid metabolism represents another important metabolic axis of gut–brain communication. PD-associated metabolomic studies consistently reveal alterations in secondary bile acids, including deoxycholic acid and lithocholic acid (47). These changes influence both peripheral and central processes: cytotoxic bile acids can exacerbate ENS inflammation, while altered signaling through bile acid receptors such as TGR5 and FXR modulates neuronal energy metabolism and neuroprotective pathways.

Collectively, these findings support a model in which gut microbiota–derived metabolites function as key intermediaries that bridge peripheral metabolic disturbances with central immune activation, thereby contributing to the initiation and propagation of neuroinflammation in PD (54).

The weight of evidence indicates that the gut–brain αSyn axis involves both retrograde and anterograde components, suggesting bidirectional rather than strictly unidirectional transmission. While the Braak hypothesis originally emphasized retrograde vagal propagation from the ENS to the brainstem, accumulating data support anterograde spread of αSyn pathology from the gut toward higher CNS structures. PD-associated gut dysbiosis, characterized by depletion of SCFA-producing taxa and enrichment of proinflammatory species, creates an intestinal environment conducive to αSyn aggregation and ENS neuron injury (18, 47). Neuronally derived extracellular vesicles carrying αSyn are detectable in the peripheral circulation of individuals at risk for developing PD, indicating that pathological αSyn species can access the systemic compartment (55). Peripheral dendritic cells in the gut-associated lymphoid tissue that encounter enteric αSyn can present epitopes via MHC-II to naïve CD4^+^ T cells in mesenteric lymph nodes, priming αSyn-specific Th1 and Th17 effector cells that subsequently traffic to the CNS through the disrupted BBB (14, 39). This peripheral immune priming cascade, initiated in the gut and amplified by systemic immune activation, thus represents a mechanistically distinct pathway through which intestinal immune dysregulation contributes to central neuroinflammation in PD, complementing direct neuronal propagation routes.

### 8.2 Astrocyte reactivity: the double-edged sword of neuroprotection and neurotoxicity

Astrocytes perform essential homeostatic functions in the central nervous system (CNS), including maintenance of BBB integrity via perivascular endfeet, regulation of synaptic glutamate levels, provision of neurotrophic factors, including BDNF and GDNF, and metabolic support to neurons through the astrocyte–neuron lactate shuttle. In PD, astrocytes undergo reactive transformation (astrogliosis), with their functional output—neuroprotective or neurotoxic—determined by the nature, intensity, and duration of upstream inflammatory signals (14).

Transcriptomic studies have identified two major reactive astrocyte states with distinct functional properties. A1 astrocytes, driven by microglia-derived cytokines, notably TNF-α, IL-1α, and C1q, exhibit a neurotoxic phenotype characterized by upregulation of complement components and loss of synaptic support functions. These cells predominate under chronic inflammatory conditions and contribute to neuronal injury. In contrast, A2 astrocytes, typically induced under ischemic or reparative conditions, upregulate neurotrophic factors such as LIF and BDNF as well as antioxidant defense pathways, thereby supporting neuronal survival and tissue repair.

Importantly, astrocyte reactivity in PD is not static but reflects a dynamic balance between these opposing states. Within the substantia nigra, a shift toward A1-dominant astrocyte populations correlates inversely with dopaminergic neuron density, suggesting that dysregulated astrocyte polarization contributes to neurodegeneration. This transition is closely linked to upstream microglial activation, positioning astrocytes as key downstream effectors within the innate immune network.

Collectively, astrocytes function as a critical interface that translates microglia-derived inflammatory signals into either neuroprotective support or neurotoxic amplification, thereby acting as a double-edged regulator of disease progression in PD.

### 8.3 Astrocyte metabolic reprogramming and inflammatory coupling

A1 reactive astrocytes reduce lactate secretion (impairing the astrocyte-neuron lactate shuttle, ANLS), diminishing neuronal energy reserves under metabolic stress. Impaired glutamate uptake (GLT-1 downregulation in A1 astrocytes) causes synaptic glutamate accumulation and excitotoxicity, further exacerbating mitochondrial dysfunction. GLP-1 receptors expressed on astrocytes allow GLP-1 receptor agonists to partially reverse A1 transformation, restoring BDNF/GDNF secretion and glutamate uptake via the cAMP/PKA-CREB pathway (56).

## 9 Lipid metabolism and the neuroinflammatory-metabolic coupling network

### 9.1 The GBA-lysosome-αSyn triangular axis

*GBA1* heterozygous mutations are the most prevalent genetic susceptibility factor for PD, with a carrier frequency of 5–15% across populations., directly establishing a causal relationship between lysosomal lipid metabolism and PD^3^. GCase functional deficiency causes lysosomal accumulation of its substrates—glucosylceramide (GluCer) and glucosylsphingosine (GluSph) (44).

Navarro-Romero et al. demonstrated that direct GluSph supplementation to wild-type cells recapitulates the complete pathological phenotype of lysosomal membrane disruption, CMA deficiency, and αSyn aggregation increase—establishing the direct toxicity of the lipid itself (not merely loss of GCase activity) (44). Lysosomal membrane disruption allows lysosomal contents (hydrolases and partially aggregated αSyn fragments) to leak into the cytoplasm, activating NLRP3 inflammasome (lysosomal rupture as a second signal) and promoting extracellular αSyn release.

GluCer accumulation also activates mTORC1 and blocks TFEB nuclear entry, globally suppressing lysosomal biogenesis and autophagy flux—a comprehensive autophagy-lysosome pathway (ALP) failure (44).

### 9.2 Sphingolipid metabolic network: disease progression metabolic signatures

Using longitudinal data from the Parkinson’s Progression Markers Initiative (PPMI) cohort, Yang et al. demonstrated that plasma ceramide-to-sphingomyelin (Cer/SM) ratios are inversely correlated with striatal dopamine transporter binding (DAT-SBR), and that baseline sphingolipid profiles can predict motor progression over a 3-year period (57). hese findings highlight sphingolipid metabolism as a clinically relevant indicator of disease progression in PD.

Within this metabolic network, ceramide occupies a central pathogenic position. At the mitochondrial level, ceramide promotes apoptosis by forming large, stable pores in the outer mitochondrial membrane, facilitating cytochrome c release and activation of the intrinsic apoptotic pathway (57). In parallel, ceramide functions as a potent signaling lipid that links metabolism to inflammation. It activates protein phosphatase 2A (PP2A), suppresses Akt/PKB survival signaling, and promotes NF-κB activation via PKCζ, thereby establishing a feed-forward loop between lipid dysregulation and inflammatory signaling.

Conversely, sphingomyelin serves as a structural and functional reservoir that buffers ceramide accumulation. Accordingly, the Cer/SM ratio can be interpreted as an index of lipid buffering capacity: a reduced ratio reflects both depletion of protective sphingomyelin and accumulation of pro-apoptotic ceramide. This imbalance is associated with increased vulnerability of nigral dopaminergic neurons and provides a mechanistic link between lipid metabolism, neuroinflammation, and neurodegeneration in PD (57).

### 9.3 CSF lipidomics: a liquid mirror of neuropathology

Fernández-Irigoyen et al.’s CSF lipidomics study (UPLC-ESI-qToF-MS/MS) identified 257 lipid species and found broadly elevated glycerolipids, phosphatidylcholines (PC), phosphatidylethanolamines (PE), ceramides, and sphingomyelins in PD patients (58). CSF lipid dysregulation degree correlates with Braak neuropathological stage and disease duration, suggesting that lipid profiles have disease staging potential.

### 9.4 Biomarkers: mechanistically grounded clinical translation

**Figure 4:**
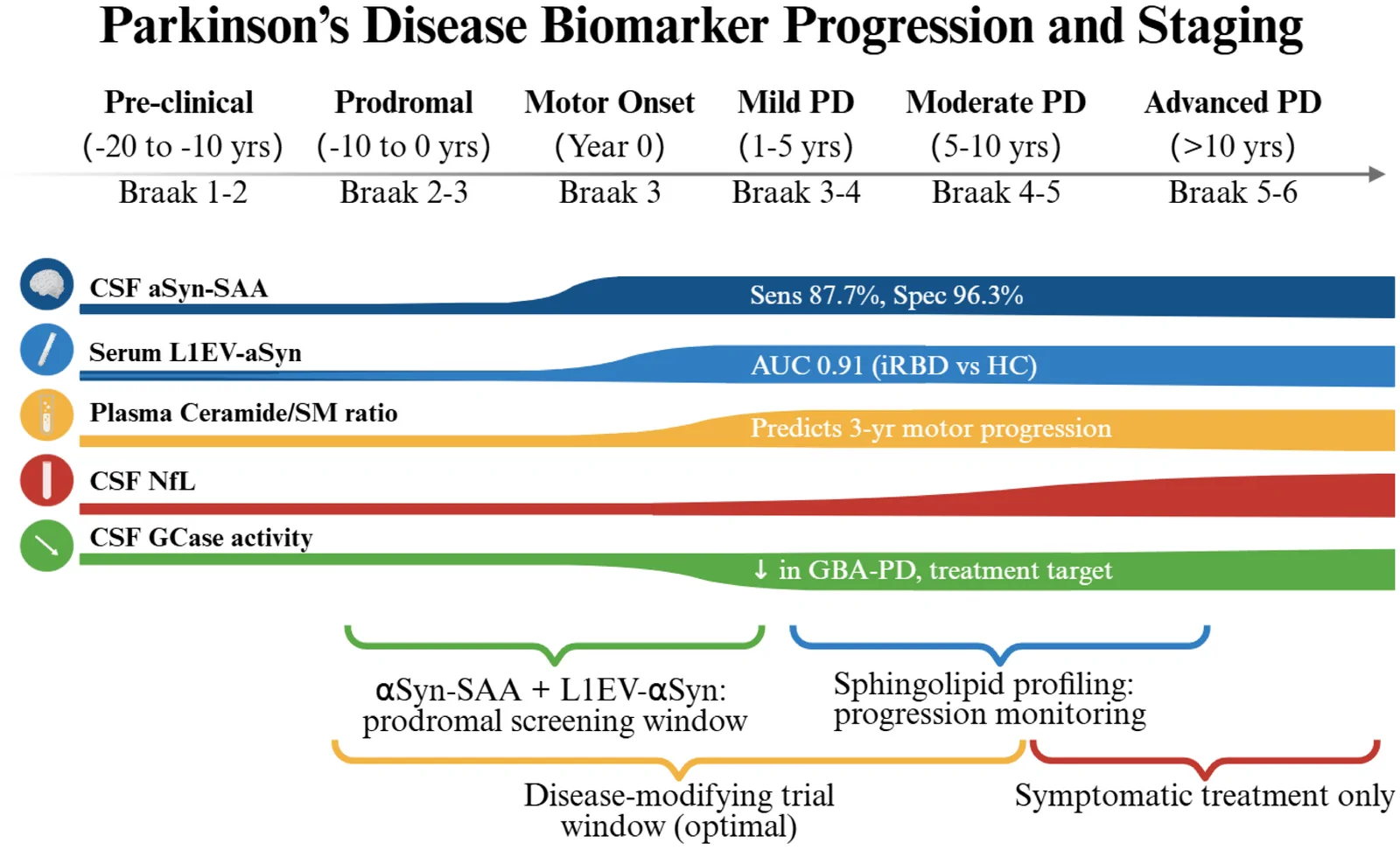
Biomarker dynamics and disease staging integration in Parkinson’s disease. Dynamic trajectories of key biomarkers across disease progression (Braak stages 1–6). CSF αSyn-SAA and serum L1EV-αSyn rise significantly in the prodromal phase (prodromal screening window). Plasma ceramide/sphingomyelin ratio predicts 3-year motor progression. CSF GCase activity progressively declines (therapeutic efficacy biomarker for GBA-targeted therapies). The optimal window for disease-modifying therapy (DMT) spans the prodromal to early motor onset stages. Created with BioRender.com.

CSF α-synuclein seed amplification assays (αSyn-SAA/RT-QuIC) represent the most robust molecular diagnostic biomarkers for PD. In the large-scale PPMI cohort (n = 3, 233), αSyn-SAA achieved 87.7% sensitivity and 96.3% specificity for sporadic PD, with positivity detected in 86% of individuals with rapid eye movement sleep behavior disorder (RBD), a high-risk prodromal population (46). These findings establish αSyn-SAA as a cornerstone tool for biological confirmation of PD pathology (**Figure 4**).

Complementing CSF-based diagnostics, neuronally derived extracellular vesicle (L1EV) α-synuclein has emerged as a promising non-invasive blood biomarker. Yan et al. validated serum L1CAM-positive EV α-synuclein across four independent cohorts, achieving an area under the curve (AUC) of 0.91 for distinguishing idiopathic RBD from healthy controls (55), highlighting its potential utility in early and prodromal disease detection.

Biomarkers reflecting lysosomal dysfunction further enable patient stratification and therapeutic monitoring. Reduced glucocerebrosidase (GCase) activity in CSF and plasma correlates with α-synuclein aggregation burden in GBA-associated PD and serves as a key pharmacodynamic endpoint in clinical trials of ambroxol and related therapies (58). In parallel, plasma neurofilament light chain (NfL), a marker of axonal injury, is increased in PD—more prominently in atypical parkinsonian syndromes—and has been validated as a predictor of disease progression in prospective cohorts such as PPMI and BioFINDER (1).

Collectively, the integration of these biomarkers enables a multidimensional precision medicine framework in PD. αSyn-SAA provides biological confirmation of underlying pathology, L1EV-associated α-synuclein supports early and prodromal screening, sphingolipid profiling informs disease progression, and GCase activity facilitates monitoring of targeted therapeutic responses (**Table 1**). Together, these tools form the core biomarker toolbox for disease stratification and individualized intervention in PD (1).

**Table 1:** Key neuroinflammatory and metabolic biomarkers in Parkinson’s disease.

Biomarker	Fluid/tissue	Mechanistic basis	Clinical utility	Key evidence
αSyn-SAA (RT-QuIC)	CSF	Prion-like αSyn seeding; reflects Lewy pathology burden	Diagnostic confirmation; prodromal detection	(46)
L1EV-αSyn	Serum	Neuronally derived EV; bypasses BBB	Non-invasive prodromal screening	(55)
GCase activity	CSF/plasma	GBA1-lysosomal axis dysfunction; drives αSyn aggregation	Pharmacodynamic endpoint for GBA-targeted therapy	(44, 61)
Ceramide/sphingomyelin ratio	Plasma	Lysosomal ceramide overload; mitochondrial membrane disruption	Progression monitoring; 3-year motor outcome predictor	(57)
Plasma NfL	Blood	Axonal degeneration marker; non-specific	Disease staging; atypical parkinsonism differentiation	(1)
Succinate	CSF/plasma	TCA immunometabolic signal; activates HIF-1α and NLRP3	Reflects microglial metabolic reprogramming state	(27)
SCFAs (butyrate, propionate)	Feces/serum	Gut microbiome-derived; epigenetically regulate microglial activation	Gut-brain axis biomarker; microbiome intervention target	(18, 47)
IL-6/TNF-α	CSF/serum	NF-κB downstream effectors of microglial activation	Neuroinflammation severity indicator	(13, 14)
Phosphatidylcholine species	Plasma/CSF	Membrane integrity; mitochondrial function reflection	Lipidomic subtyping; treatment response monitoring	(16, 66)
Mitochondrial DNA (mtDNA)	CSF/plasma	mtDAMP; activates cGAS-STING and TLR9 pathways	Neuroinflammation trigger biomarker	(25)

Biomarkers are grouped by biological fluid/compartment. SAA, seed amplification assay; EV, extracellular vesicle; GCase, glucocerebrosidase; NfL, neurofilament light chain; SCFA, short-chain fatty acid; S/SM, ceramide-to-sphingomyelin ratio; DMT, disease-modifying therapy; BBB, blood-brain barrier; RCT, randomized controlled trial; AUC, area underthe ROC curve.

## 10 Therapeutic perspectives: immunometabolic axis-targeting strategies

### 10.1 GLP-1 receptor agonists: bridging metabolic protection and neuroinflammation

Repurposing glucagon-like peptide-1 receptor agonists (GLP-1RAs) represents one of the most clinically translatable strategies in PD immunometabolic therapy. This approach is supported by substantial mechanistic overlap between PD and type 2 diabetes mellitus (T2DM), such as insulin resistance, mitochondrial dysfunction, neuroinflammation, and α-synuclein aggregation (59). Epidemiological evidence consistently supports this link: meta-analyses have shown that individuals with T2DM face an approximately 32% increased risk of developing PD, and those who develop the disease exhibit more severe and rapidly progressing phenotypes.

At the molecular level, GLP-1RAs exert neuroprotective effects through coordinated actions across multiple cell types, converging on shared immunometabolic pathways. In neurons, GLP-1R activation stimulates the cAMP–PKA–CREB signaling cascade, leading to upregulation of anti-apoptotic proteins such as Bcl-2 and neurotrophic factors including BDNF, while suppressing JNK-dependent apoptotic signaling. Concurrently, GLP-1RAs restore mitochondrial function by improving complex I activity, reducing mtROS production, and preserving mitochondrial membrane potential in toxin-induced PD models (60).

In the context of neuroinflammation, GLP-1RAs attenuate major inflammatory signaling cascades by inhibiting NF-κB nuclear translocation and suppressing NLRP3 inflammasome assembly, thereby reducing the production of proinflammatory cytokines, including TNF-α, IL-1β and IL-6. These effects are accompanied by enhanced microglial autophagic clearance of α-synuclein aggregates, linking metabolic modulation to improved proteostasis (60). In addition, GLP-1R signaling in astrocytes promotes a shift away from neurotoxic A1 phenotypes toward a more supportive state, restoring the secretion of neurotrophic factors such as BDNF and GDNF through cAMP-dependent mechanisms (56).

Collectively, these findings position GLP-1RAs as key modulators of the immunometabolic network in PD, simultaneously targeting neuronal survival, mitochondrial integrity, inflammatory signaling, and glial function. This multi-level convergence highlights their potential as disease-modifying therapies that bridge metabolic intervention with neuroimmune regulation.

### 10.2 AMPK/mTOR axis: endogenous metabolic reprogramming targets

AMPK activators (metformin, AICAR, resveratrol) simultaneously promote mitochondrial biogenesis (PGC-1α), activate mitophagy (clearing damaged mitochondria), inhibit mTORC1, and attenuate NF-κB signaling, demonstrating dopaminergic neuroprotection in PD cell and animal models (22).

### 10.3 GBA-targeted strategies: lysosomal-lipid axis intervention

Targeting glucocerebrosidase (GCase) dysfunction represents a central therapeutic strategy aimed at restoring the lysosomal–lipid axis in PD. Reduced GCase activity results in glucosylceramide accumulation, impaired lysosomal degradation, and enhanced α-synuclein aggregation, establishing a pathogenic feedback loop that links lipid metabolism to proteostasis failure. Accordingly, current GBA-targeted interventions can be broadly categorized into pharmacological chaperones, substrate reduction strategies, and gene therapy approaches.

Pharmacological chaperones such as ambroxol aim to stabilize misfolded GCase and enhance its lysosomal trafficking and activity. Phase II clinical studies have demonstrated that ambroxol effectively penetrates the central nervous system, increases CSF GCase activity, and reduces α-synuclein levels in both GBA-associated and sporadic PD patients. However, a recent Phase II trial of ambroxol in PDD did not meet its primary cognitive endpoint, indicating that the therapeutic effects of GCase chaperoning may be context-dependent and require careful patient selection. These mixed results highlight the need for further mechanistic stratification and the development of more potent GCase-targeting compounds (61).

Substrate reduction therapy represents an alternative strategy to rebalance sphingolipid metabolism upstream of GCase. Venglustat, an inhibitor of glucosylceramide synthase (GCS), reduces the synthesis of glucosylceramide and has been investigated in Phase II clinical trials in GBA-associated PD (44).

Gene therapy approaches seek to restore GCase activity at its source. Adeno-associated virus serotype 9 (AAV9)–based delivery of GBA1 is currently under evaluation in early-phase clinical trials, offering the potential for sustained correction of lysosomal dysfunction.

Collectively, these strategies target complementary levels of the lysosomal–lipid axis—enhancing enzyme function, reducing substrate burden, and restoring gene expression—highlighting a convergent therapeutic framework aimed at interrupting the lipid–α-synuclein pathogenic cycle in PD.

### 10.4 Gut microbiome modulation

Prebiotic/dietary fiber supplementation to restore SCFA levels, specific probiotic strains showing motor function improvement in PD animal models, and fecal microbiota transplantation (FMT) reporting constipation improvement in PD patients—all represent therapeutic strategies targeting the gut-brain immunometabolic axis (47).

### 10.5 Research gaps and future directions

Substantial advances have been made in understanding PD immunometabolic mechanisms. However, several critical gaps remain that limit both mechanistic understanding and clinical translation (**Table 2**).

**Table 2:** Immunometabolic axis-targeting therapeutic strategies in Parkinson’s disease.

Drug/intervention	Target/pathway	Mechanism of action	Development stage	Key findings/evidence
Exenatide	GLP-1 receptor	Reduces neuroinflammation and supports mitochondrial function and proteostasis.	Phase II and Phase III. The earlier Phase II signal was not confirmed in Exenatide-PD3; the Phase III primary endpoint was not met.	(63)
Semaglutide	GLP-1 receptor	Combines metabolic protection with anti-inflammatory and pro-autophagic effects.	Phase II ongoing. Disease-modifying efficacy in PD remains unestablished.	(56, 67)
Metformin	AMPK/mTOR/NF-κB	Activates AMPK and suppresses mTOR- and NF-κB-associated signaling.	Observational and Phase II evaluation. Current clinical signals remain hypothesis-generating.	(22, 59, 67)
Rapamycin/analogues	mTORC1	Restores autophagy and mitophagy through mTORC1 inhibition.	Preclinical/early translational development. Clinical efficacy and long-term tolerability in PD are unknown.	(17, 67)
Ambroxol	GCase/GBA1 pathway	Acts as a GCase chaperone and enhances lysosomal trafficking and target engagement.	Phase II; larger trials ongoing. CNS penetration and target engagement were demonstrated, but a PDD trial did not improve primary cognitive outcomes.	(44, 61)
Venglustat	GBA1/glucosylceramide synthase	Reduces glucosylceramide synthesis upstream of the GCase pathway.	Phase II (MOVES-PD). Central target engagement occurred, but primary and secondary clinical efficacy outcomes showed no benefit.	(65)
MCC950/analogues	NLRP3 inflammasome	Blocks NLRP3 activation, IL-1β/IL-18 maturation, and pyroptotic signaling.	Preclinical. Neuroprotective effects are reported in models; human PD efficacy is untested.	(68)
Fecal microbiota transplantation	Gut microbiome/gut–brain axis	Attempts to restore microbial and short-chain fatty acid homeostasis.	Pilot clinical studies. Larger controlled trials with standardized protocols are required.	(69)
LRRK2 kinase inhibitors	LRRK2/Rab/lysosomal pathway	Reduces LRRK2-dependent Rab hyperphosphorylation and supports lysosomal trafficking.	Phase I/II. Target engagement and early safety have been evaluated; efficacy remains under study.	(67)
NAD^+^ precursors	NAD^+^/SIRT1/PGC-1α	Supports mitochondrial quality control and restrains NF-κB signaling.	Phase I/II. Biomarker and feasibility studies are underway; clinical efficacy is not established.	(67, 70)

MOA, mechanism of action; RCT, randomized controlled trial; T2DM, type 2 diabetes mellitus; LRRK2, leucine-rich repeat kinase 2; GBA, glucocerebrosidase; mTOR, mechanistic target of rapamycin; NF-κB, nuclear factor kappa B; NLRP3, NOD-, LRR-, and pyrin domain-containing protein 3; IND, investigationalnew drug. Drugs are grouped by primary target/pathway.

First, the causal relationship between metabolic dysregulation and PD pathology remains unresolved. It is still unclear whether lipid metabolic alterations act as upstream drivers of α-synuclein aggregation or arise as downstream consequences of neurodegeneration. More broadly, the causal architecture linking neuroinflammation and metabolic dysfunction itself remains to be formally adjudicated: longitudinal studies are needed to determine whether these processes are directly coupled, share a common upstream driver, or represent parallel downstream sequelae of a primary neurodegenerative process. Emerging multi-omics Mendelian randomization studies provide initial genetic evidence supporting potential causal links, but further validation is required (62).

Second, the optimal therapeutic window for immunometabolic intervention is not well defined. While preclinical studies suggest that early modulation of microglial metabolism may confer greater neuroprotection, clinical implementation has been limited by delayed diagnosis. The development of prodromal biomarkers, including αSyn-SAA and neuron-derived extracellular vesicle α-synuclein (L1EV-αSyn), now offers the opportunity to initiate intervention at earlier disease stages and design prevention-oriented clinical trials (46).

Third, PD exhibits substantial metabolic heterogeneity across disease subtypes. Distinct forms such as LRRK2-associated PD, GBA-associated PD, and sporadic PD differ in α-synuclein biology, inflammatory profiles, and metabolic signatures. These differences reinforce the importance of trial designs with molecular stratification and precision medicine approaches tailored to subtype-specific pathophysiology (46).

Fourth, sex differences in PD remain underexplored. Epidemiological data indicate a higher prevalence in males (approximately 1.5-fold compared to females), while emerging evidence suggests sex-specific differences in lipid metabolism and mitochondrial resilience. However, the mechanistic basis of these differences and their therapeutic implications are not yet fully understood.

Finally, current metabolomic approaches lack sufficient resolution to capture cell type–specific metabolic dynamics. The integration of single-cell and spatial metabolomics, together with high-resolution imaging platforms such as stimulated Raman scattering (SRS) microscopy, is expected to enable precise mapping of metabolic states across distinct neural and immune cell populations.

Collectively, addressing these challenges will be pivotal for translating immunometabolic discoveries into actionable disease-modifying therapies and for advancing a precision medicine framework in PD.

## 11 Animal models and clinical evidence: translational validation

### 11.1 Preclinical models

Preclinical models provide essential platforms for validating the immunometabolic mechanisms underlying PD and for establishing causal relationships between metabolic dysfunction and neuroinflammation. These models can be broadly categorized into neurotoxin-based, α-synuclein propagation, and genetic models, each recapitulating distinct aspects of disease pathophysiology.

Neurotoxin models—including MPTP, rotenone, and 6-hydroxydopamine (6-OHDA)—have been extensively employed to investigate mitochondrial dysfunction–driven neuroinflammation. In the MPTP model, its active metabolite MPP^+^ blocks mitochondrial complex I, resulting in elevated mtROS production, assembly of the NLRP3 inflammasome, and secretion of proinflammatory cytokines, including TNF-α and IL-1β. This process is accompanied by enhanced microglial glycolytic reprogramming. Importantly, genetic deletion of NLRP3 or pharmacological activation of AMPK via metformin pretreatment significantly attenuates MPTP-induced nigral degeneration, providing direct evidence of a causal contribution of the NLRP3–metabolic axis to PD pathology (22, 60).

The rotenone model further supports the integration of mitochondrial dysfunction, α-synuclein pathology, and systemic involvement. Chronic systemic rotenone delivery in rodent models reproduces core PD features, including selective dopaminergic neuron loss, Lewy body–like α-synuclein inclusions, and enteric nervous system pathology, consistent with the gut-origin hypothesis. Metabolomic profiling in this model reveals upregulation of sphingolipids and downregulation of phosphatidylcholine species, closely mirroring plasma metabolic signatures observed in PD patients and validating the translational relevance of lipid dysregulation (16, 37).

α-Synuclein preformed fibril (PFF) injection models provide a complementary platform to study prion-like propagation of pathology. Stereotaxic injection of PFFs into the striatum induces progressive, Braak-like spread of α-synuclein aggregates. Notably, these models enable precise temporal resolution of disease progression: microglial metabolic reprogramming occurs early (within 1–2 weeks), preceding detectable neuronal loss; CSF α-synuclein seed amplification assays become positive at intermediate stages (4–6 weeks); and peak nigral dopaminergic degeneration is observed at later time points (8–12 weeks). This temporal sequence supports the hypothesis that metabolic reprogramming is an upstream driver of neurodegeneration (35).

Genetic models, including LRRK2 G2019S, PINK1^−^/^−^, Parkin^−^/^−^, and GBA N370S variants, further reveal genotype-specific immunometabolic mechanisms. For example, PINK1^−^/^−^ mice exhibit relatively mild baseline mitochondrial dysfunction but display exaggerated neuroinflammatory responses upon immune challenge, such as lipopolysaccharide (LPS) exposure. These findings suggest that mitochondrial dysfunction primarily lowers the threshold for inflammatory activation rather than directly inducing chronic inflammation.

Collectively, these preclinical models converge on a unified framework in which mitochondrial dysfunction, metabolic reprogramming, and innate immune activation form a causally linked network driving PD pathology (23).

### 11.2 Key randomized controlled clinical trials

Recent randomized controlled trials (RCTs) provide emerging clinical evidence supporting immunometabolic intervention strategies in PD, particularly targeting metabolic–inflammatory pathways.

Among these, GLP-1 receptor agonists (GLP-1RAs) have generated the most compelling signals. In a randomized, double-blind, placebo-controlled Phase II trial, exenatide demonstrated a sustained improvement in motor function, with an approximately 7-point between-group difference in MDS-UPDRS part III scores at the end of a 12-week washout phase following 48 weeks of treatment (56). The persistence of benefit beyond treatment cessation suggests potential disease-modifying effects rather than purely symptomatic relief.

Subsequent trials have yielded more nuanced results. The Liraglutide Phase II trial (LIRA-PD), involving 100 participants over 52 weeks, did not meet its primary endpoint based on total MDS-UPDRS scores. However, subgroup analyses revealed stronger therapeutic signals in patients with baseline metabolic syndrome and in male participants, indicating that metabolic phenotype and sex may influence responsiveness to GLP-1RA therapy (56). These findings support the concept that patient stratification based on metabolic status may be critical for optimizing treatment efficacy.

The importance of patient stratification is further underscored by the recent Phase III Exenatide-PD3 trial (63), which failed to replicate the earlier Phase II positive findings in a broader PD cohort, with the primary endpoint not reaching statistical significance. Together, the negative outcomes of LIRA-PD and Exenatide-PD3 suggest that unselected patient populations may obscure potentially beneficial effects that emerge only in metabolically defined subgroups. This highlights the necessity of integrating biomarker-guided stratification and optimized dosing regimens into future GLP-1RA trial designs.

Ongoing studies continue to refine this therapeutic class. The Semaglutide Phase II trial (SPARK-PD), a multicenter UK RCT with approximately 120 participants and a 104-week follow-up, is designed to evaluate long-term disease-modifying effects. Semaglutide offers potential advantages, including higher central nervous system exposure and convenient once-weekly dosing, which may improve both efficacy and adherence (64).

Beyond GLP-1RAs, strategies targeting the GCase pathway have also undergone clinical validation. The MOVES-PD trial, which evaluated the GCase substrate reduction therapy venglustat in GBA mutation carriers, was terminated early due to failure to meet its primary endpoint. This negative outcome further highlights the necessity of precision patient screening within genetically defined subtypes and the challenges of translating preclinical GCase-modulating strategies into clinical benefits (65).

In parallel, therapeutic strategies targeting upstream inflammatory signaling are entering early clinical development. Inzomelid-class selective small-molecule NLRP3 inflammasome inhibitors have progressed to Phase I safety evaluation in neurodegenerative disorders., supported by robust neuroprotective effects observed in MPTP and α-synuclein preformed fibril (PFF) models (14).

Collectively, these clinical trials highlight a translational trajectory in PD therapeutics, moving from metabolic modulation (GLP-1RAs) toward direct targeting of inflammatory hubs such as NLRP3. Importantly, the variability in clinical outcomes underscores the need for biomarker-guided patient stratification and precision trial design to fully realize disease-modifying potential.

### 11.3 Biomarker integration in clinical trial design

The integration of molecular and metabolic biomarkers into clinical trial design represents a critical step toward precision medicine in PD. Biomarkers can be deployed across multiple stages of trial architecture, including patient selection, stratification, pharmacodynamic monitoring, and target prioritization.

At the level of patient selection, α-synuclein seed amplification assays (αSyn-SAA) provide a robust biological confirmation criterion that enables exclusion of non–α-synuclein parkinsonian phenocopies. This is particularly relevant given that a substantial proportion of genetically defined cases—such as approximately one-third of LRRK2-associated PD—are αSyn-SAA–negative, highlighting the importance of mechanistic stratification to improve cohort homogeneity (46).

For patient stratification and treatment monitoring, pathway-specific biomarkers offer additional granularity. Glucocerebrosidase (GCase) activity serves both as an enrichment criterion and as a primary pharmacodynamic endpoint in trials targeting the lysosomal–lipid axis, including ambroxol and venglustat studies (58). In parallel, plasma ceramide-to-sphingomyelin (Cer/SM) ratios provide a metabolomic indicator of disease progression rate, enabling identification of patient subgroups with more aggressive trajectories for inclusion in progression-sensitive trials (57).

Beyond individual biomarkers, integrative genomic approaches are increasingly informing target selection. Multi-omics Mendelian randomization analyses by Li et al, have identified over 140 candidate therapeutic genes enriched in immune–metabolic pathways, supported by multilayer genetic evidence (62). These data provide a rational framework for prioritizing targets in future mechanism-based clinical trials.

Collectively, these advances support a biomarker-driven trial design paradigm in which biological confirmation, molecular stratification, dynamic monitoring, and genetically informed target selection are integrated to enhance trial efficiency and increase the likelihood of detecting disease-modifying effects in PD.

## 12 Conclusion

This review has systematically examined the mechanistic coupling between neuroinflammation and metabolic dysfunction in Parkinson’s disease. We propose that these two processes may function not as independent parallel pathologies, but as components of a mutually reinforcing pathological network through multilayered molecular dialogue. However, this integrative model should be interpreted with caution. The available evidence does not exclude the possibility that neuroinflammation and metabolic dysfunction arise from a common upstream driver, such as mitochondrial dysfunction or genetic susceptibility, rather than being directly coupled. Alternatively, they could represent parallel downstream sequelae of a primary neurodegenerative process. Distinguishing these alternatives will require longitudinal studies with repeated multi-omics measurements and causal inference approaches. Microglial metabolic reprogramming from OXPHOS to glycolysis is simultaneously the energetic prerequisite for NF-κB/NLRP3 signal amplification and the metabolic root of diminished αSyn clearance capacity; mitochondrial dysfunction-derived mtROS directly injures neurons while activating cGAS-STING through mtDNA mislocalization to reshape the CNS inflammatory landscape; lysosomal lipid dysregulation (GBA-sphingolipid axis) both drives αSyn aggregation and amplifies neuroinflammation through inflammasome activation; and reduced gut microbiome-derived SCFAs simultaneously compromises intestinal barrier integrity and ENS homeostasis while directly modulating central microglial inflammatory thresholds through epigenetic mechanisms.

This integrated framework carries direct translational implications: it explains why single-target interventions targeting αSyn alone or inflammation alone have consistently failed to demonstrate sustained disease-modifying efficacy—the self-amplifying nature of the disease demands coordinated intervention at multiple nodes. GLP-1 receptor agonists are particularly noteworthy in this context, as their mechanism simultaneously integrates metabolic protection (mitochondrial function restoration, insulin signal improvement) and anti-inflammation (NF-κB/NLRP3 inhibition), acting on neurons, microglia, and astrocytes across three critical cell types (56, 60).

Looking ahead, as prodromal biomarkers such as αSyn-SAA advance toward clinical deployment, single-cell multi-omics technologies mature, and molecularly stratified precision trial designs proliferate, PD neuroinflammation-metabolomics research stands poised to transition from mechanistic discovery toward actionable clinical intervention. Positioning metabolomics and lipidomics as the “translational language” connecting molecular mechanisms with clinically measurable phenotypes will be the core methodological framework driving the next breakthrough in this field.
